# Correction: Yukhnovets et al. Impact of Molecule Concentration, Diffusion Rates and Surface Passivation on Single-Molecule Fluorescence Studies in Solution. *Biomolecules* 2022, *12*, 468

**DOI:** 10.3390/biom14081002

**Published:** 2024-08-14

**Authors:** Olessya Yukhnovets, Henning Höfig, Nuno Bustorff, Alexandros Katranidis, Jörg Fitter

**Affiliations:** 1 AG Biophysik, I. Physikalisches Institut (IA), RWTH Aachen University, 52074 Aachen, Germany; h.hoefig@fz-juelich.de; 2 Ernst Ruska-Centre for Microscopy and Spectroscopy with Electrons (ER-C-3), Institute of Biological Information Processing IBI-6, Forschungszentrum Jülich, 52425 Jülich, Germany; n.bustorff@fz-juelich.de (N.B.); a.katranidis@fz-juelich.de (A.K.)

In the original publication [1], there was a mistake in Table 1 as published. In Section 2.10, the values for the molar concentration in Table 1 were miscalculated by a factor of two for V ≈ 0.5 fL and 1 fL, as well as calculated with the wrong V-values for V ≈ 2 fL and 4 fL. The corrected Table 1 appears below. Since the values in Table 1 only serve to illustrate the general relationship between detection volume, particle number, and molar concentration, all further results and conclusions in the paper are not influenced by this.

The authors state that the scientific conclusions are unaffected. This correction was approved by the Academic Editor. The original publication has also been updated.

## Figures and Tables

**Table 1:** Comparison of different molar molecule concentrations as determined from *N* for different confocal detection volumes.

Pinhole Ø	30 μm	50 μm	75 μm	150 μm
	^1^ V ≈ 0.5 fL	V ≈ 1 fL	V ≈ 2 fL	V ≈ 4 fL
*N*	Molar Concentration in pM			
0.001	3.32	1.66	0.83	0.42
0.005	16.61	8.30	4.15	2.08
0.01	33.21	16.61	8.30	4.15
0.02	66.42	33.21	16.61	8.30
0.03	99.63	49.82	24.91	12.45
0.05	166.06	83.03	41.51	20.76
0.1	332.12	166.06	83.03	41.51

^1^ Effective detection volumes depend on the excitation and fluorescence emission wavelengths. The values given here represent a mean value, as obtained by averaging from 485 and 640 nm wavelength conditions.
