# Supplementary material for: Impact of Molecule Concentration, Diffusion Rates and Surface Passivation on Single-Molecule Fluorescence Studies in Solution
Source: Biomolecules. 2022 Mar 18;12(3):468. doi: 10.3390/biom12030468 (PMC8946791; doi:10.3390/biom12030468)
Supplement: Supplementary file 1 [file biomolecules-12-00468-s001.zip › biomolecules-1609749-supplementary.pdf]

S 1 Brightness-Gated Two Color Coincidence Detection

Brightness-gated two color coincidence detection (BTCCD) is a method that is used to quantify the binding fraction in a bi-molecular reaction on single-molecule level. It utilizes simultaneous two-color confocal detection that has an inherent problem of incomplete overlap of confocal volumes for different excitation wavelengths (Figure S1). This leads to an underestimation of the coincidence fraction, due to having molecule trajectories that do not cross both detection volumes. Figure S2 shows an inter-photon lag (IPL) trace of an almost 100% double-labeled DNA. If detection volumes in both channels were fully overlapping, all bursts would appear in both, red and blue, channels after the initial burst threshold is applied to differentiate bursts from the background. This is not the case for burst I, as the molecule trajectory did not touch the blue volume. For burst II, the molecule trajectory went through the red volume and only slightly touched the blue volume. Finally, for burst III, the molecule trajectory went through both, the red and the blue volume. The idea of brightness-gating is to select only bursts for the analysis, that touched both volumes. One of the possible selection criteria that we implement is a number of photons per burst. The distribution of number of photons per burst (Figure S3) demonstrates that peripheral trajectories contain smaller number of photons, but are detected much more often than central trajectories bursts that contain high number of photons.

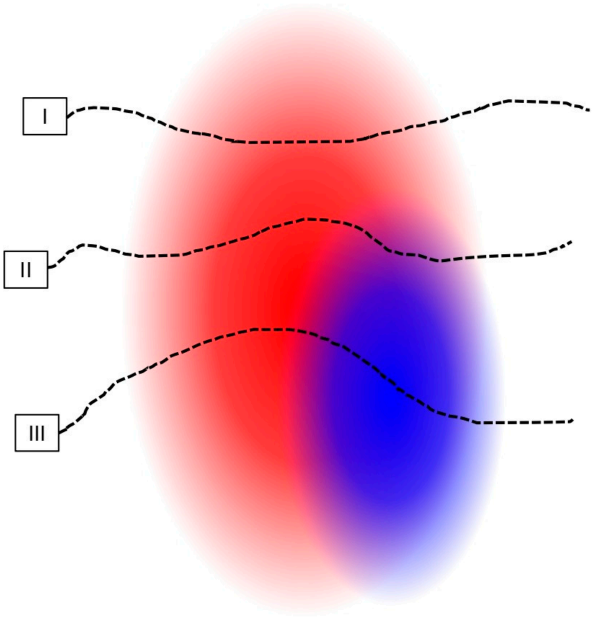

Figure S1. Schematic image of incomplete confocal detection volumes overlap for the red and the blue channels. Possible molecular trajectories are shown: I and II - peripheral, III - central.

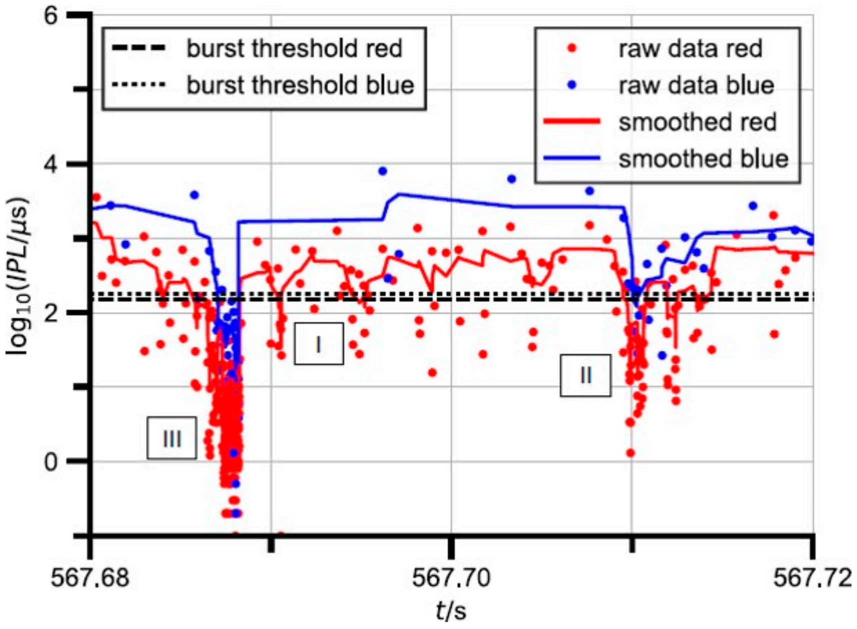

Figure S2. Illustration of burst that correspond to peripheral (I and II) and central (III) trajectories.

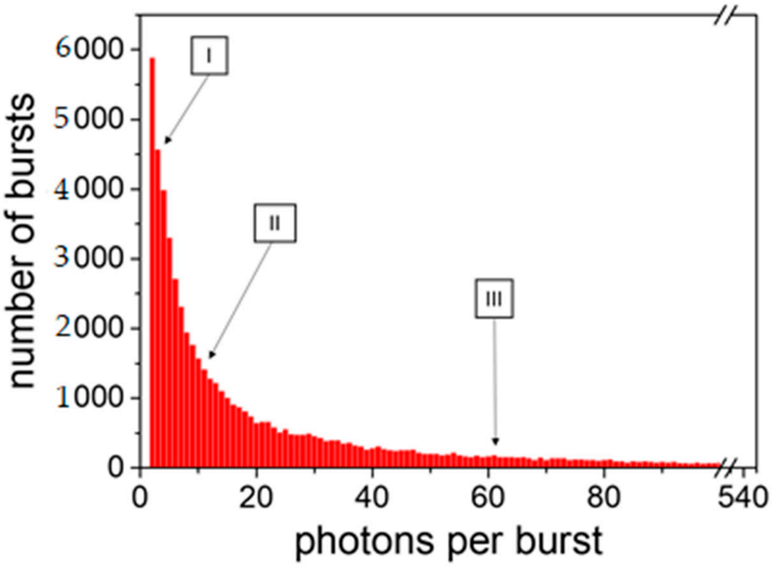

.

Figure S3. Number of photons per burst distribution for the red channel. Peripheral trajectories I and II contain a smaller number of photons compared to the central trajectories III.

It is obviously impossible to define the fixed threshold number of photons value at which only central molecule trajectories will be selected. This value will depend on the excitation power, photophysical properties of dyes and possible local quenching and is calculated separately for each channel. The brightness threshold selects only bursts that contain more photons than selected by the threshold value. As this value can also be different for both channels, the brightness threshold, normalized to the mean number of photons is given by:

$$n_{br} = \frac{n_{photons}}{< n_{photons} >} \qquad (1)$$

Coincidence values are calculated separately for red and blue channels:

$$f_{RB}(n_{br}) = \frac{N_{RB}(n_{br})}{N_R(n_{br})}, \qquad (2a)$$

$$f_{BR}(n_{br}) = \frac{N_{BR}(n_{br})}{N_B(n_{br})}, \qquad (2b)$$

n_{br} is continuously increased until the coincidence fraction is constant and all peripheralbursts are excluded and the plateu is reached (Figure S4).

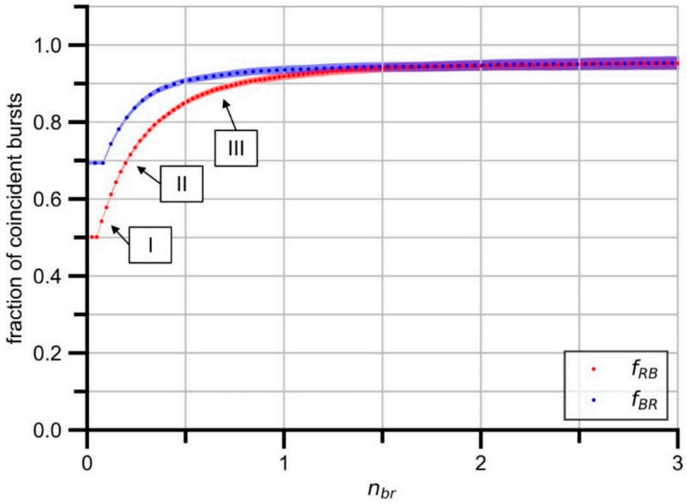

Figure S4. Coincidence fractions of dsDNA (Alexa 488/Atto 647N) as a function of the bright- ness threshold. The shaded area represents the statistical error. The contribution of peripheral bursts at low *nbr* values results in the underestimation of the coinci- dence fraction (trajectory I). Gradual exclusion of peripheral bursts (trajectory II) shows an increase in the coincidence fraction until it plateaus at higher *nbr* values (trajectory III).

Detailed description of the BTCCD method can be found in [1].

S 2 Chance Coincidence Calculation

The content presented in this section was taken from the PhD thesis of H. Höfig [2]. The basis of a single-molecule experiment is that the probability for the presence of more than one molecule at a time in the detection volume is negligible. In a FRET experiment, multi-molecule events lead to a loss of the single-molecule information or even to a misinterpretation of the data [3]. In a BTCCD experiment, multi-molecule events lead accordingly to an artificial increase of the coincidence fractions because chance coincidences of singlelabeled molecules are interpreted as dual-labeled molecules. Obviously, the impact of chance coincidences is more pronounced if the true coincidence fractions are anyhow low.

The probability of multi-molecule events is negligible if the average number of molecules in the detection volume is $N \leq 0.03$. However, multi-molecule events can become significant if a brightness gating is applied. This effect was reported for single-molecule FRET experiments in which a threshold for the donor and acceptor photon counts is applied [3]. For BTCCD the same effect is present. The probability of chance coincidences is increased for large brightness thresholds because bright bursts have also a longer dwell time and, consequently, it is more likely that during this increased dwell time another molecule enters the detection volume. In the following, an expression for the probability of chance coincidences is derived for single-labeled molecules. The description is extended to dual-labeled molecules in the following section.

First, only one species of molecules is considered. The probability that during an observation time t a number of n single molecules enter the detection volume is given by a Poisson distribution [4]

$$p_n^e(t, \tau_d, N) = \frac{(Nt/\tau_d)^n}{n!} exp(-Nt/\tau_d), \tag{3}$$

where N is the average number of molecules in the detection volume and τd is the mean dwell time in the volume. With the definition of a mean time for entering the volume $\tau e = \tau d / N$, equation 3 simplifies to

$$p_n^e(t, \tau_e) = \frac{(t/\tau_e)^n}{n!} exp(-t/\tau_e). \tag{4}$$

The probabilities for no entry ($n = 0$) and one entry ($n = 1$) are consequently:

$$p_0^e(t, \tau_e) = exp(-t/\tau_e) \tag{5a}$$
$$p_1^e(t, \tau_e) = t/\tau_e \, exp(-t/\tau_e) \tag{5b}$$

Assuming a constant dwell time, the probability for the occurrence of a burst caused by n single molecules can be estimated based on the following idea. Several bursts caused by multiple molecules cannot be resolved if the molecules enter during a time which is faster than the dwell time. Consequently, the probability pmn of a multi-molecule burst caused by n molecules is given by the product of the probabilities of n−1 molecules

entering successively with a delay less than the dwell time τd and by the probability that before and after that sequence there is no molecule in the volume [5]:

$$
\begin{aligned}
pm_n(N) &= p_0^e(\tau_d, \tau_e)\left[\prod_{j=1}^{n-1} p_1^e(t_j < \tau_d, \tau_e)\right] p_0^e(\tau_d, \tau_e) \\
&= exp(-\tau_d/\tau_e)\left[\prod_{j=1}^{n-1}\int_0^{\tau_d}\frac{dt_j}{\tau_e} exp(-t_j/\tau_e)\right] exp(-\tau_d/\tau_e) \\
&= exp(-2N)(1 - exp(-N))^{n-1}
\end{aligned} \tag{6}
$$

Equation 6 shows that the probability of multi-molecules events depends only on the average number of molecules in the detection volume. The relevant probability for a chance coincidence event is pmn=2 because the presence of two molecules at a time in the detection volume is the minimal requirement for a chance coincidence:

$$pm_{n=2}(N) = exp(-2N)(1 - exp(-N)) \tag{7}$$

All other probabilities pmn>2 lead also to chance coincidences but their probabilities are smaller and can be neglected for $N \ll 1$. Finally, not the absolute probability to observe a chance coincidence is of interest but the ratio to the probability to observe a single-molecule event:

$$f_{mm} = \frac{pm_{n=2}(N)}{pm_{n=1}(N)} = 1 - exp(-N) \tag{8}$$

Is there a threshold value for N above which multi-molecule events become considerable? The answer on this questions depends obviously on the error one want to tolerate. If a fraction of multi-molecules events of 1% can be tolerated, a critical mean number of molecules Nc is given by

$$f_{mm} = 0.01 \iff 1 - exp(-N_c) = 0.01 \iff N_c = -ln(0.99) \approx 0.01\,. \tag{9}$$

Chance coincidences in BTCCD

In BTCCD only cross-color multi-molecule events need to be considered. If a red-labeled molecule is located in the detection volume, a chance coincidence occurs when a blue-labeled molecule enters the volume during the dwell time of the red

molecule. The probability for entering of a blue-labeled molecule during the dwell time of a red-labeled molecule can be calculated in analogy to Equation 5b and 6

$$p_e^{RB} = p_1^e(t < \tau_d^R, \tau_e^B) = 1 - exp\left(-\left(\tau_d^R/\tau_d^B\right) N_B\right) , \quad (10)$$

where $\tau R d$ and $\tau B d$ are the mean dwell times of the red-labeled and blue-labeled molecules, respectively, and NB is the average number of blue-labeled molecules in the detection volume. Instead of entering after the red-labeled molecule, the blue-labeled molecule could also be located in the volume before the red-labeled molecule entered. Consequently, both probabilities, i.e. blue-labeled molecule already present or entering during dwell time of red molecule, have to be added and it needs to be considered that just red-only molecules lead to a falsepositive event. Hence, the fraction of chance coincidence is given by

$$\begin{aligned} f_{chance}^{RB} &= (1 - f_{RB}) \left[exp(-N_B)\left(1 - exp\left(-\left(\tau_d^R/\tau_d^B\right) N_B\right)\right) + 1 - exp(-N_B)\right] \\ &= (1 - f_{RB}) \left[1 - exp\left(-N_B\left(\left(\tau_d^R/\tau_d^B\right) + 1\right)\right)\right] \end{aligned} \quad (11)$$

where fRB is the fraction of dual-labeled red molecules. An analogue expression can be derived for $f\ BR$ $chance$ if blue and red is interchanged in Equations 10 and 11:

$$f_{chance}^{BR} = (1 - f_{BR}) \left[1 - exp\left(-N_R\left(\left(\tau_d^B/\tau_d^R\right) + 1\right)\right)\right] \quad (12)$$

Equations 11 and 12 show a few implications that arise for cross-color multi-molecule events: (i) The fraction of chance coincidences in the red channel depends on the ratio of the dwell times of the red and blue labeled molecules. The larger the red dwell time, the more probable it is that a blue molecule enters within that time and, accordingly, the lower the blue dwell time, the more frequently blue molecules will enter the detection volume. By applying the brightness threshold to the red channel, the red dwell time will increase whereas the blue dwell time stays constant. Consequently, the fraction of chance coincidences increases for increasing brightness thresholds. (ii) The fraction of chance coincidences in the red channel depends only on the mean number of blue molecules because red-red coincidences are not considered. However, red-red coincidences lead to an apparent increase of the red dwell time which is neglected here. (iii) The impact of chance coincidences is less pronounced if the molecules have already a high fraction of dual labeling. A dual labeled molecule cannot be misinterpreted by a chance coincidence. Again, the same conclusions can be drawn for chance coincidences of the blue channel.

Equations 11 and 12 are only an approximation because a constant dwell time and a perfect overlap of the blue and red detection volumes were assumed.

S 3 Concentration stability for PGK smFRET measurements for different GuHCl concentrations

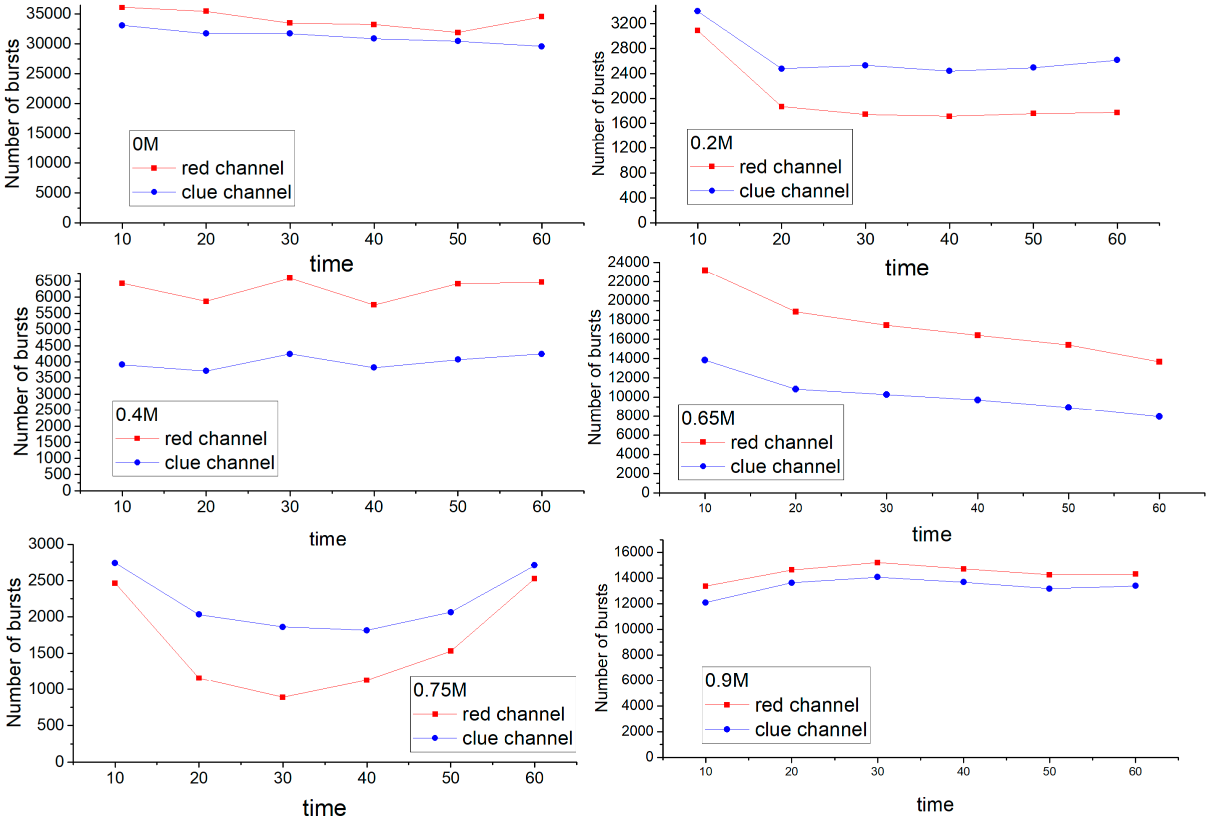

Figure S5. Number of bursts change over one hour measurement time for different GuHCl concentration. Number of bursts remains more or less stable for all concentrations of GuHCl except for 0.65M and 0.75M (transition state of PGK).

S 4 smFRET 2D plot for dsDNA mix

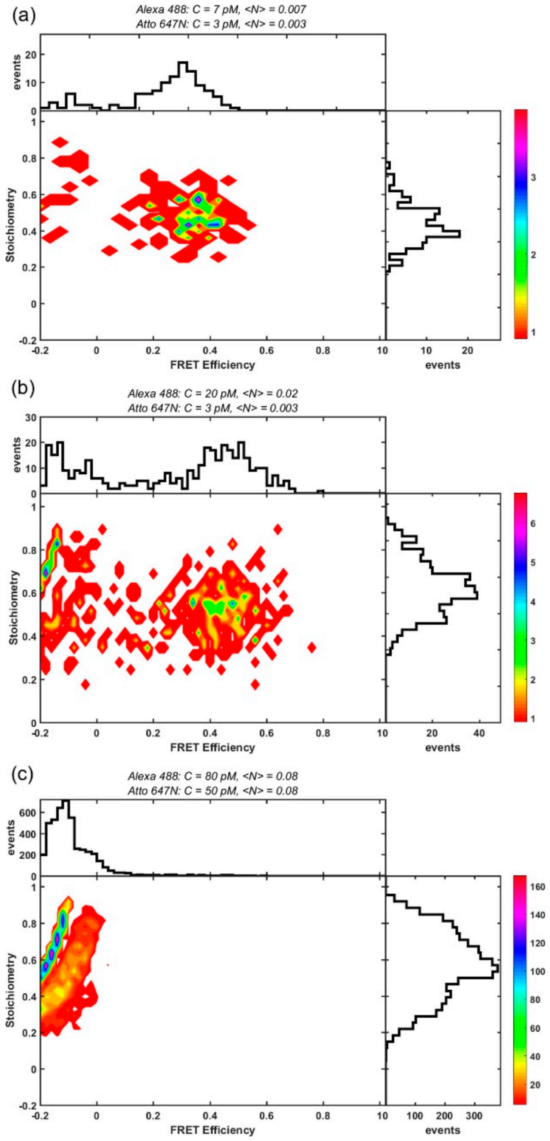

Figure S6. smFRET 2D plot of dsDNA mix of 3% Alexa 488/Alexa 647 labeled, 48.5% Alexa 488 labeled, 48.5% Alexa 647 labeled to demonstrate the importance of purification for samples with low double-labeling yield (a) no free dye, FRET population is clearly seen despite low percentage of double-labeled species (b) by adding 13 pM of free Alexa 488, FRET population starts to merge slightly with the E = 0 peak (c) by adding 73 pM of free Alexa 488 and 47 pM of free Atto 647N, the FRET population disappears as most of the time there is more than one molecule in the detection volume and the probability to detect single double-labeled molecule is very low. The scenarios presented in panel a and c are rather similar to those shown in Figure 5a and 5b in the main manuscript.

References

[1] Henning Höfig et al. “Brightness-gated two-color coincidence detection unravels two distinct mechanisms in bacterial protein translation initiation”. In: *Communications biology* 2.1 (2019), pp. 1–8.

[2] Henning Höfig. “Single-Molecule characterization of FRET-based biosensors and development of Two-Color coincidence detection”. PhD thesis. Dissertation, RWTH Aachen University, 2020.

[3] Irina V Gopich. “Concentration effects in “single-molecule” spectroscopy”. In: *The Jour- nal of Physical Chemistry B* 112.19 (2008), pp. 6214–6220.

[4] Lars Edman, Ulo Mets, and Rudolf Rigler. “Conformational transitions monitored for single molecules in solution”. In: *Proceedings of the National Academy of Sciences* 93.13 (1996), pp. 6710–6715.

[5] Jörg Enderlein et al. “Molecular shot noise, burst size distribution, and single-molecule detection in fluid flow: effects of multiple occupancy”. In: *The Journal of Physical Chemistry A* 102.30 (1998), pp. 6089–6094.
